# Long-term outcome in new onset refractory status epilepticus: a retrospective study

**DOI:** 10.1186/s13054-024-04858-7

**Published:** 2024-03-12

**Authors:** Federica Stretti, Stefan Yu Bögli, Francesca Casagrande, Amanda Eisele, Marian Galovic, Emanuela Keller, Giovanna Brandi

**Affiliations:** 1 Institute of Intensive Care Medicine, University Hospital Zurich, Rämistrasse 100, 8091 Zurich, Switzerland; 2 Department of Neurology, Clinical Neuroscience Center, University Hospital Zurich, Frauenklinikstrasse 26, 8091 Zurich, Switzerland; 3 Department of Neurosurgery and Clinical Neuroscience Center, University Hospital and University of Zurich, Frauenklinikstrasse 26, 8091 Zurich, Switzerland; 4 University of Zurich, Zurich, Switzerland

**Keywords:** New onset refractory status epilepticus, Status epilepticus, End of life, Outcome, Modified Rankin scale

## Abstract

**Background:**

New onset refractory status epilepticus (NORSE) is a neurologic emergency without an immediately identifiable cause. The complicated and long ICU stay of the patients can lead to perceiving a prolongation of therapies as futile. However, a recovery is possible even in severe cases. This retrospective study investigates ICU treatments, short- and long-term outcome and ethical decisions of a case series of patients with NORSE.

**Methods:**

Overall, 283 adults were admitted with status epilepticus (SE) to the Neurocritical Care Unit of the University Hospital Zurich, Switzerland, between 01.2010 and 12.2022. Of them, 25 had a NORSE. We collected demographic, clinical, therapeutic and outcome data. Descriptive statistics was performed.

**Results:**

Most patients were female (68%), previously healthy (Charlson comorbidity index 1 [0–4]) and relatively young (54 ± 17 years). 96% presented with super-refractory SE. Despite extensive workup, the majority (68%) of cases remained cryptogenic. Most patients had a long and complicated ICU stay. The in-hospital mortality was 36% (*n* = 9). The mortality at last available follow-up was 56% (*n* = 14) on average 30 months after ICU admission. The cause of in-hospital death for 89% (*n* = 8) of the patients was the withholding/withdrawing of therapies. Medical staff except for one patient triggered the decision. The end of life (EOL) decision was taken 29 [12–51] days after the ICU admission. Death occurred on day 6 [1–8.5] after the decision was taken. The functional outcome improved over time for 13/16 (81%) hospital survivors (median mRS at hospital discharge 4 [3.75–5] vs. median mRS at last available follow-up 2 [1.75–3], *p* < 0.001).

**Conclusions:**

Our data suggest that the long-term outcome can still be favorable in NORSE survivors, despite a prolonged and complicated ICU stay. Clinicians should be careful in taking EOL decisions to avoid the risk of a self-fulfilling prophecy. Our results encourage clinicians to continue treatment even in initially refractory cases.

## Background

New onset refractory status epilepticus (NORSE) is a neurologic emergency in patients without a previous history of epilepsy and without an immediately identifiable underlying cause [1, 2]. At least 50% of patients with NORSE remain cryptogenic despite extensive diagnostic workup [3]. Consequently, NORSE presents not only diagnostic but also therapeutic challenges as well as ethical dilemmas. The severity of NORSE necessitates sedation and invasive treatments, which is why patients usually need to be admitted to the Intensive Care Unit (ICU); the ICU stay is complicated and extremely long, ranging from weeks to months [4–7].

The extensive use of resources and the uncertainty of outcome can lead the treating physicians to perceive a prolongation of therapies as futile. Status epilepticus (SE) carries a mortality of more than 15% [8]. NORSE, a subset of SE, is usually also thought to have a poor outcome, but data in the literature are scarce. Mortality might be lower than in other forms of SE, possibly due to the absence of structural brain injury that independently affects outcome [3]. Functional outcome, additionally, has been even less investigated: In the few available studies, it has been reported to improve over time and to be good in more than 50% of the survivors [3, 6, 9].

NORSE usually affects previously healthy and young individuals, and a recovery is possible even in severe and prolonged cases. Factors found to affect outcome are age [6], duration of SE and etiology [3], but these data remain necessarily anecdotal. Due to the rarity of the disease and the geographical spread of the cases, there is, to date, no controlled prospective large study of NORSE that could help to generalize results. Consequently, no validated prognostic tools are available that could help physicians and families in the difficult decision-making process of setting the boundaries between beneficence and non-maleficence. On the one side, treatment can be continued in view of a possible good recovery, as opposed to the other side, when the prolongation of maximal treatment can be deemed as futile.

To bridge this gap in knowledge, this retrospective study investigates patients with NORSE admitted to the neurological ICU of a university hospital in Switzerland over twelve years. We aimed at addressing ICU intensity of treatment, complications and outcome of patients, with a particular focus on ethical decisions at the end of life (EOL).

## Methods

### Study population and data collection

We retrospectively screened adults (≥ 18 years old) admitted with SE to the Neurocritical Care Unit (NCCU) of the University Hospital Zurich, Switzerland, between January 2010 and December 2022. We identified patients with NORSE according to current consensus definitions [1]. Data were retrospectively collected from the medical records of the included patients. We collected demographic, clinical, diagnostic, therapeutic and outcome parameters from medical records. NORSE etiology was defined based on a multimodal diagnostic assessment, including a magnetic resonance imaging (MRI). At our hospital, the MRI sequences for status epilepticus are diffusion-weighted (DWI), susceptibility-weighted (SWI), T1- and T2-weighted, 3D FLAIR and T1 3D eventually with contrast medium. Outcome data (modified Rankin scale (mRS)) were assessed at discharge, at 12 months and at last available follow-up for survivors.

### Standard protocol approvals, registrations and patient consents

Patients were excluded from the study in case of written or documented oral refusal to have their data analyzed for research projects. The local ethic committee (Kantonale Ethikkommission Zürich, KEK) approved the study (BASEC2020-02880), which was performed in accordance with the ethical standards as laid down in the 2013 Declaration of Helsinki. This manuscript adheres to the applicable STROBE guidelines.

### Statistical analysis

Statistical analysis was performed using SPSS version 26. Descriptive statistics are reported as counts/percentages, mean ± standard deviation, or as median including the interquartile range as appropriate. All continuous data were tested for normality using Shapiro–Wilk's test. Data not normally distributed were compared using the Mann–Whitney test. A *p* value < 0.05 was considered significant. Numerical variables with normal distribution were compared using independent sample t test. Ordinal variables or numerical variables with not normal distribution were compared using Mann–Whitney-Wilcoxon test. Categorical variables were compared with chi-squared test.

## Results

### Baseline characteristics and ICU stay/treatment

Of the 283 patients with SE admitted to our ICU between January 2010 and December 2022, 25 met the criteria for NORSE and were included in the study. These patients were relatively young (age 54 ± 17 years), previously healthy based on the Charlson Comorbidity Index (1 [0–4]) and mostly female (68%). The demographics and patients’ characteristics are outlined in Tables 1 and 2.

**Table 1:** Demographics and patients’ characteristics (*n* = 25 patients)

Age, in years (SD)	54 (17)
Female *n* (%)	17 (68)
Charlson Comorbidity Index [IQR]	1 [0–4]
mRS at admission to hospital [IQR]	3 [2–5]
STESS	3 [2–4]
SOFA	8 [5–9]
SAPS	48 [36–58]
Super-refractory SE, *n* (%)	24 (96)
Cause of NORSE identified, *n* (%)	8 (32)
MRI performed, *n* (%)	25 (100)
Lumbar puncture performed, *n* (%)	25 (100)
FDG-PET	13 (52)
Brain biopsy	5 (20)
Reasons for ICU admission	
Seizures, *n* (%)	13 (52)
Coma, *n* (%)	7 (28)
Confusion/Agitation, *n* (%)	3 (12)
Others, *n* (%)	2 (8)

*SE* Status epilepticus; *mRS* modified Rankin scale; *STESS* Status Epilepticus Severity Score; *SOFA* Sequential Organ Failure Assessment Score; *SAPS* Simplified Acute Physiology Score II; *NORSE* New onset refractory status epilepticus. *MRI* Magnetic resonance; *FDG-PET* 18-Fluorodeoxyglucose-positron emission tomography; *ICU* Intensive care unit. Data are present as counts/percentages, mean ± standard deviation (SD), or as median including the interquartile range (IQR), as appropriate

**Table 2:** Detailed demographic data and scores per patient

Patient	Sex	Age (years)	CCI	SAPS II	mRS at adm	STESS score	SE duration (days)	Level of consciousness at admission
1	F	41	0	31	2	1	49	Confusion
2	F	72	10	58	5	4	20	Coma
3	M	67	4	36	5	4	20	Coma
4	F	46	0	35	2	4	4	Stupor
5	F	59	2	52	3	1	19	Coma
6	F	77	8	74	5	5	15	Coma
7	F	40	0	32	3	2	18	Confusion
8	F	82	9	62	5	6	4	Coma
9	F	72	5	58	2	2	15	Stupor
10	F	55	1	21	5	4	20	Somnolence
11	M	67	10	48	5	0	32	Coma
12	F	53	2	39	2	3	7	Somnolence
13	M	49	0	19	2	3	4	Coma
14	M	66	2	42	2	1	4	Confusion
15	F	32	0	45	5	5	8	Coma
16	M	76	4	70	1	2	9	Coma
17	F	53	0	33	3	5	6	Stupor
18	F	49	0	54	3	2	91	Coma
19	M	74	3	64	0	6	24	Coma
20	F	61	17	50	3	5	6	Stupor
21	F	35	0	36	5	2	13	Stupor
22	F	33	0	48	5	3	14	Somnolence
23	M	41	0	64	5	3	71	Coma
24	F	31	0	63	5	3	12	Coma
25	M	17	0	57	4	3	3	Somnolence

*CCI* Charlson comorbidity index; SAPS II, Simplified Acute Physiology Score; mRS at adm,, modified Rankin score at hospital admission; STESS, Status Epilepticus Severity Score; SE, status epilepticus

The majority of patients (96%) presented with super-refractory SE and needed a combination of antiepileptic drugs (AED) (median number of AEDs 5 [4-7]) as well as sedatives to control the SE. Despite extensive workup (100% lumbar puncture, 100%, magnetic resonance imaging), the cause for NORSE could be identified in only 32% of the patients. Data on diagnostics and imaging are shown in Table 3, as well as description of semiology of SE, electroencephalogram (EEG) findings, use and duration of continuous EEG. Overall, 18 patients (72%) received a continuous EEG, all of them for at least 2 days.

**Table 3:** Diagnostics and imaging

Patient	NORSE Etiology	WCC (pro µL)	Albumin quotient in CSF	Abnormalities at first MRI	Brain FDG-PET abnormalities	First EEG	Origin or maximum of ED/SE	EEG at ICU-discharge or last EEG before death	Origin or maximum of epileptic discharges	Semiology of seizures	cEEG	Duration of continuous EEG (days)	In-hospital death	Timing of death (days after hospital admission)
1	Crypto	8	N	No	Heterogeneous cortical hyper- and hypometabolism	DB	NA	DB	NA	Repetitive GTCS	Yes	45	Yes	52
2	CASPR-2 AI encephalitis	2	N	DWI restriction: left parahippocampal gyrus	NA	MFS/SE	O	MFS/SE	O	One GTCS, then NC	Yes	19	Yes	34
3	CAS with recurrent perfusion deficit	29	N	DWI restriction: cortical, bilateral, parietal	NA	FS/SE	TP	No ED, no SE	NA	One GTCS, then focal or NC	No	NA	No	–
4	Crypto	276	↑	No	No	FS/SE	F	Repetitive, focal ED	F	NC	Yes	3	No	–
5	Crypto	4	↑	T2-/FLAIR- Hyperintensities, white substance mainly left occipitotemporal	NA	MFS/SE	FC	No ED, no SE	NA	NC	No	NA	No	–
6	Crypto	0	NA	LE	NA	FS/SE	F	FSE	F	NC	Yes	8	Yes	41
7	Crypto	0	N	T2-/FLAIR- Hyperintensities right insula, cingulate gyrus, hippocampus	No	MFS/SE	F	No ED, no SE	NA	Repetitive GTCS	No	NA	No	–
8	Crypto	32	NA	LE	NA	FS/SE	F	Repetitive, focal ED	T	One GTCS, then NC	No	NA	No	743
9	Crypto	5	N	DWI restriction, right thalamus	No	FS/SE	F	Few, focal ED	TP	Repetitive GTCS	Yes	15	No	–
10	Crypto	6	↑	T2-/FLAIR Hyperintensities cortical frontal, cingulate gyrus, thalamus, cerebellar bilateral	No	FS/SE	F	NA	NA	Repetitive GTCS	Yes	16	No	995
11	CJD	1	NA	No	No	FS/SE	C	FS or SE	O	Focal	Yes	5	Yes	32
12	HSV-1	23	↑	CE right temporal	NA	FS/SE	T	Repetitive, generalized ED	F	NC	No	NA	Yes	8
13	Tick-borne encephalitis	130	↑	T2-/FLAIR-hyperintensities right parietooccipital	NA	MFS/SE	F	No ED, no SE	NA	Repetitive GTCS	No	NA	No	–
14	HSV type 1	53	N	T2-/FLAIR- Hyperintensities right temporal and insular	NA	FS/SE	T	No ED, no SE	NA	NC	Yes	10	No	676
15	Crypto	20	NA	T2-/FLAIR- Hyperintensities in the basal ganglia	NA	FS/SE	C	NA	NA	Repetitive GTCS	Yes	2	Yes	9
16	Crypto	19	N	LE	NA	FS/SE	F	No ED, no SE	NA	Two GTCS, then NC	Yes	2	Yes	70
17	HSV-1	626	↑	T2-/FLAIR- Hyperintensities left temporal, amygdala and hippocampus	NA	FS/SE	T	No ED, no SE	NA	One GTCS, then NC	Yes	4	No	–
18	Crypto	34	N	T2-/FLAIR- Hyperintensities putamen and nucleus caudatus bilateral	Generalized hypometabolism	MFS/SE	TF	FS	TP	Focal	Yes	80	No	–
19	Crypto	1	N	DWI restriction, global	NA	FS/SE	PO	Repetitive, focal ED	P	NC	Yes	21	Yes	59
20	Crypto	1	↑	No	No	MFS/SE	F	No ED, no SE	NA	NC	No	NA	No	82
21	Crypto	7	↑	DWI restriction. FLAIR-hyperintensities left hippocampus	No	MFS/SE	F	Few, multifocal ED	F	Repetitive GTCS	Yes	17	No	–
22	Anti-GABAA receptor encephalitis	0	N	FLAIR- Hyperintensities temporal, amygdala, hippocampus, gyrus cinguli	Metabolically active amygdala and hippocampus on the left, most likely inflammatory	MFS/SE	T	FS	C	Focal	Yes	19	No	–
23	Crypto	27	N	No	No	MFS/SE	F	Multifocal periodic ED, no SE	F	Focal	Yes	79	No	531
24	Crypto	28	↑	FLAIR- hyperintensity, punctiform precentral right	No	MFS/SE	C	No ED, no SE	NA	Repetitive GTCS	Yes	34	Yes	192
25	Crypto	12	↑	No	No	FS/SE	T	Multifocal periodic ED, no SE	TP	Repetitive GTCS	Yes	22	No	–

*AI* Autoimmune, *C* Central; *CAS* Carotid artery stenosis; *CE* Contrast enhancement; *CJD* Creutzfeldt-Jakob disease, *CJD* Crypto, cryptogenic; *DB* Delta brushes; *ED* Epileptic discharges; *F* Frontal; *FC* Frontocentral; *FS* Focal seizures; *GTCS* Generalized tonico-clonic seizures; *LE* Leucoencephalopathy; *MFS* Multifocal seizures; *N* Normal; *NA* Not available; *NC* Nonconvulsive; *PO* Parietooccipital; *O* occipital; *SE* Status epilepticus; *T* Temporal; *TF* Temporofrontal; *TP* Temporoparietal; ↑, Elevated

The main reason for the ICU admission was seizures (52%), followed by coma (28%). Most patients had a long and complicated stay in the ICU, requiring invasive ventilation (96%), vasopressors (72%) and tracheostomy (60%), as shown in Table 4. All patients developed at least one complication and a third of them more than one. The most frequent complications were infections (72%) and respiratory (64%). A summary of the complications is shown in Table 4.

**Table 4:** Treatment and complications

Number of AEDs (median[IQR])	5 [4–7]
Duration of treatment with anesthetic agents (days, median[IQR])	8 [6–18]
Need of invasive mechanical ventilation, *n* (%)	24 (96)
Duration of invasive mechanical ventilation (days, median[IQR])	17 [9–25]
Need of vasopressor support, *n* (%)	18 (72)
Duration of vasopressor support (days, median[IQR])	7 [1–16]
Need of tracheostomy, *n* (%)	15 (60)
ICU complications, *n* (%)	25 (100)
Neurological, *n* (%)	18 (72)
Delirium	5
Neurocognitive deficits	13
Focal deficits	3
CIP/CIM	9
Infections, *n* (%)	18 (72)
VAP	13
UTI	8
CRBSI	2
Respiratory, *n* (%)	16 (64)
Aspiration pneumonia	6
ARDS	2
Atelectasis	3
Others	6
Metabolic, *n* (%)	14 (56)
Gastrointestinal, n (%)	10 (40)
Cardiac, *n* (%)	9 (36)
Arrhythmia	8
NSTEMI	1
Length of stay on ICU (days, median[IQR])	21 [18–31]
Length of stay in hospital (days, median[IQR])	39 [26–55]

*AED* Antiepileptic drugs; *EEG* Electroencephalogram; *ICU* Intensive care unit

Data are present as counts/percentages, mean ± standard deviation (SD), or as median including the interquartile range (IQR), as appropriate. CIP/CIM critical illness polyneuropathy/ myopathy; VAP ventilator-associated pneumonia; UTI urinary tract infection; CRBSI catheter-associated bloodstream infection; ARDS acute respiratory distress syndrome; NSTEMI non-ST-elevation myocardial infarction

### Outcome and EOL decision

**Fig. 1:**
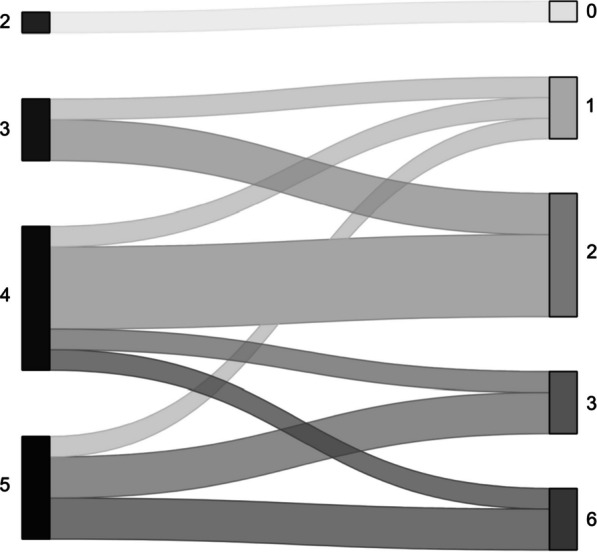
Alluvial plot on functional outcome of hospital survivors (*n* = 16) at hospital discharge (on the left side) and at the last available follow-up (on the right side). The functional outcome of hospital survivors improved over time (median mRS at hospital discharge 4 [3.75–5] vs. median mRS at last available follow-up 2 [1.75–3], *p* < 0.001). mRS Modified Rankin scale. Data are presented as median including the interquartile range (IQR)

Approximately one-third of the patients died during the hospital stay (*n* = 9, 36%) (Fig. 1). Of them, 5 died during SE. The cause of in-hospital death was for most of the patients a redirection of care to palliation (withholding/withdrawing of therapies, 8/9 patients, 89%). One patient died because of abdominal compartment syndrome. Only 2/9 patients had a written advance care directive (AD). In almost all the patients, the decision was triggered by medical staff (89%), and only in one patient by the relatives. The EOL decision was taken on day 29 [12–51] after the ICU admission and death occurred 6 [1–8.5] days after the decision was taken. The data regarding outcome are summarized in Tables 5 and 6.

**Table 5:** Outcome and end of life process

In hospital death, *n* (%)	9 (36)
Death at last available follow-up, *n* (%)	14 (56)
Limitation of treatment in deceased patients (data available for 12/14 pts), *n* (%)	10 (77)
Presence of written advance directives in deceased patients, (data available for 12/14 pts), *n* (%)	3 (25)
Trigger of EOL decision, data for patients who died in hospital *n* = 9	
Medical staff, *n* (%)	8 (89)
Relatives, *n* (%)	1
Time of EOL decision from ICU admission, days (*n* = 10)	29 [12–51]
Time from EOL decision to death, days, (*n* = 10)	6 [1–8.5]
mRS at hospital discharge (*n* = 25, median and range)	5 [4-6]
mRS at hospital discharge for survivors (*n* = 16)	4 [3.75–5]
mRS at 12 months for survivors (data available for 14/16 patients)	2 [1.25–2.75]
mRS at last available follow-up for survivors (*n* = 16; median and range)	2 [1.75–3]
Favorable mRS (0–2) at last available follow-up for hospital survivors (*n* = 16) *n* (%)	10 (62.5)
Timing of last available follow-up for hospital survivors (*n* = 16) from admission date (days, median and IQR)	728 [521–997]

*EOL* End of life; *mRS* modified Rankin scale; *ICU* intensive care unit. Data are present as counts/percentages, or as median including the interquartile range (IQR), as appropriate

**Table 6:** Functional outcome at different time points

Pat. ID	Cause of NORSE	ICU-LOS (days)	mRS at hospital admission	mRS at hospital discharge	Reason of death	mRS at 12 months	Last available mRS	Days after ICU admission of the last available mRS
1	Cryptogenic	49	2	6	Abdominal compartment syndrome with MOF	x	x	x
2	CASPR-2-antibody encephalitis	15	5	6	WLST	x	x	x
3	Recurrent deficit perfusion due to ICA stenosis	21	5	4		2	2	327
4	Cryptogenic	25	2	4		1	1	1872
5	Cryptogenic	32	3	5		1	1	740
6	Cryptogenic	41	5	6	WLST	x	x	x
7	Cryptogenic	26	3	3		2	2	4166
8	Cryptogenic	10	5	5		5	6	743
9	Cryptogenic	28	2	4		2	2	456
10	Cryptogenic	34	5	5		n.a	3	130
11	CJD	19	5	6	WLST	x	x	x
12	Herpes encephalitis	7	2	6	WLST	x	x	x
13	Tick-borne encephalitis	2	2	2		1	0	1693
14	Herpes encephalitis	20	2	4		3	3	658
15	Cryptogenic	9	5	6	WLST	x	x	x
16	Cryptogenic	20	1	6	WLST	x	x	x
17	Herpes encephalitis	20	3	3		1	1	1272
18	Cryptogenic	86	3	5		4	3	905
19	Cryptogenic	27	0	6	WLST	x	x	x
20	Cryptogenic	3	3	5		x	6	17
21	Cryptogenic	18	5	4		2	2	716
22	Anti-GABAA receptor encephalitis	21	5	3		2	2	791
23	Cryptogenic	88	5	4		4	6	531
24	Cryptogenic	191	5	6	WLST	x	x	x
25	Cryptogenic	23	4	4		2	2	550

Functional outcome. *NORSE* New onset refractory status epilepticus; *ICU-LOS* Length of stay at the intensive care unit; *mRS* modified Rankin scale; *ICA* Internal carotid artery; *CDJ* Creutzfeldt-Jakob disease; *n.a* Not available. *MOF* Multiorgan failure; *WLST* Withdrawal of life-sustaining treatment; *ICU-LOS* Length of stay at the intensive care unit

**Fig. 2:**
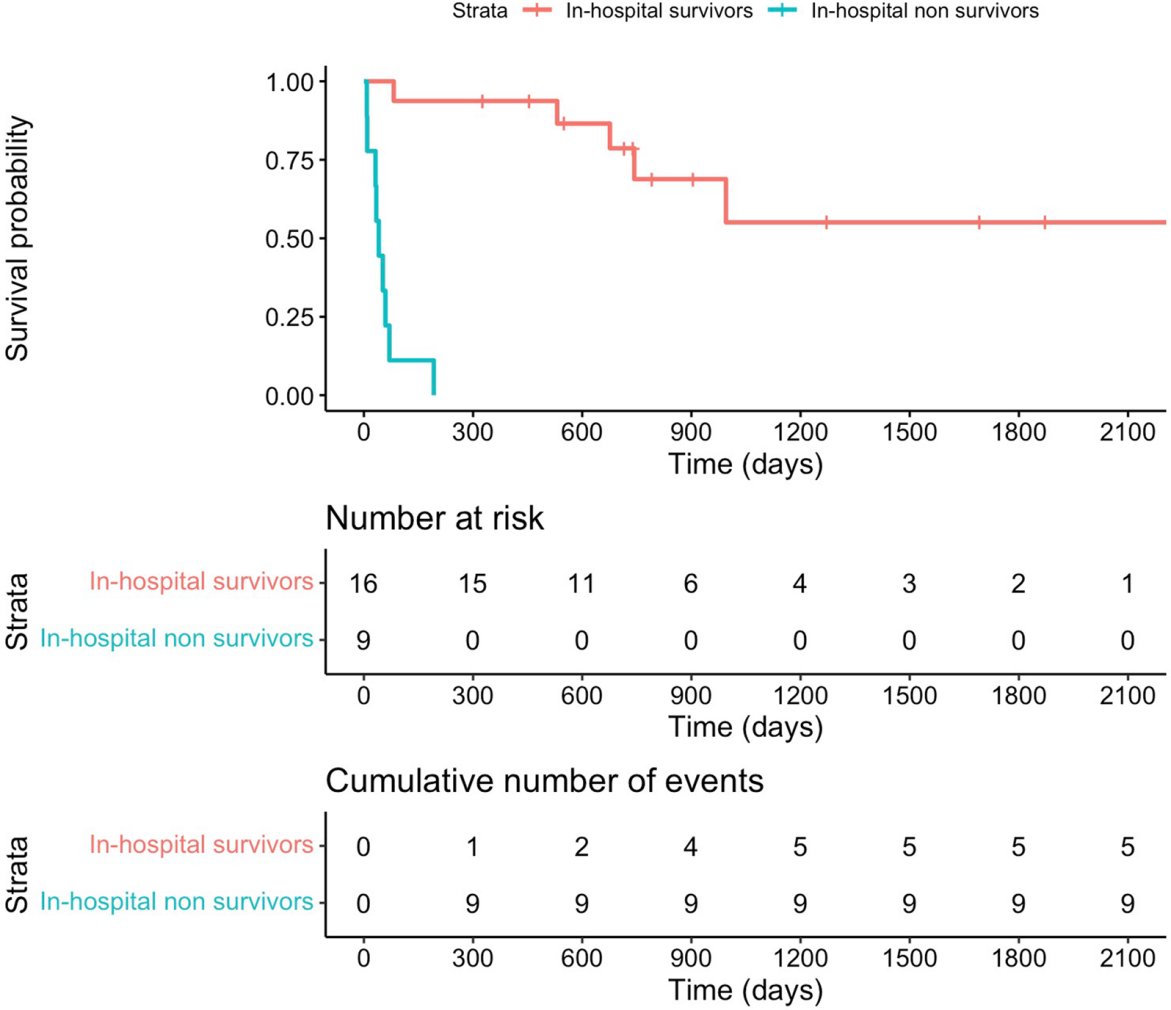
Kaplan–Meier survival curve. The red curve refers to in-hospital survivors, the blue one to in-hospital non-survivors. On the *y*-axis is indicated the survival probability, on the *x*-axis the time, as expressed in days

The functional outcome was assessed at hospital discharge, at 12 months (± 2 months) and up to 11 years after ICU admission. The functional outcome of hospital survivors improved over time (median mRS at hospital discharge 4 [3.75–5] vs. median mRS at last available follow-up 2 [1.75–3], *p* < 0.001), as shown in Fig. 1 and in Table 6. Timing of last available follow-up for hospital survivors (*n* = 16) from admission date was at a median of 728 days [521–997] (Fig. 2).

### Comparisons in-hospital survivors versus in-hospital non-survivors

In-hospital survivors (*n* = 16) and in-hospital non-survivors (*n* = 9) did not differ in the baseline characteristics (sex, age, Charlson comorbidity index), severity scores (SAPS II, modified Rankin scale at hospital admission, STESS score), as well as duration of SE and ICU length of stay (data not shown).

## Discussion

NORSE is a rare and relatively new condition, formally defined in 2018 [2]. First guidelines for the treatment of NORSE have only recently been published [10]. Because data on the disease are scarce and there is a lack of standardized protocols [11, 12], its diagnosis and management are mostly based on expert opinions.

We conducted this study to describe ICU intensity of treatment, complications, short- and long-term outcome of a cohort of patients with NORSE, with particular focus on ethical decisions at the EOL. In the study population, patients had a long ICU and hospital stay, needing invasive treatments and developing many complications. The in-hospital mortality was high, reaching 36%. On the one hand, this percentage is comparable with the scarce data from the literature in which it ranges from 22 to 42% [3, 4, 6, 9]. On the other hand, a decision to limit life-sustaining therapies (LST) was taken more often than in few previous studies [3, 6, 9]. We can think of two explanations for this. Firstly, the other studies did not specifically address this issue, so the prevalence could be underreported. Secondly, this study was performed in a university hospital in Switzerland, where the culture regarding patients’ autonomy and medical decision is peculiar. The legal framework bases therapies on patients’ will: After multidisciplinary discussion and involving the patients’ family, LSTs are deemed potentially inappropriate or futile. The decision to limit LSTs is then based on the assumption that they would result in an undesired outcome, not respecting the patients’ will. Futility cannot be objectively defined and is highly dependent on an individual’s values. Due to the neurological impairment, patients are incapable of giving informed consent and discussing prognosis and therapeutic options. As far as we can presume from the few available written ADs or from the conversations with surrogate decision makers (SDMs), most individuals perceived severe disability as an undesirable outcome and life-sustaining therapies therefore as futile. In these cases, the therapy was redirected and the patient subsequently died. Family, where present, was always involved, so it is safe to assume that the EOL decisions were informed and based on presumed patient’s wishes.

The additional challenge in the NORSE patient population, compared to other diseases, is the lack of reliable data on long-term functional outcome and of validated prognostic tools, as is the case, as an example, for traumatic brain injury [13]. The risk of self-fulfilling prophecy is high. Although many patients at discharge from hospital had an unfavorable functional status as expressed by the mRS, the functional outcome of the survivors improved consistently over time and it was favorable in the majority of them at the last available follow-up.

The decision to limit therapies was taken relatively late (median 29 days after ICU admission). For NORSE patients, a longer period of observation and treatment might be necessary: Firstly, many investigations/exams are needed and the turnover time of these usually takes many days (e.g. genetic testing, autoimmune testing). Secondly, no validated prognostic tools exist as for other diseases, which makes the prognostication harder and mandates even more an interprofessional shared decision making to avoid the risk of a self-fulfilling prophecy.

Because the median long-term outcome was favorable in the majority of NORSE patients (62.5% with mRS 0-2) and comparable to previous NORSE cohorts with lower mortality rates, our results may encourage clinicians to continue treatment even in initially refractory cases.

### Limitations

Our conclusions are limited by the study’s retrospective nature and by the fact that it is a single-center study and consequently not representative of the country as a whole. Furthermore, outcome was retrospectively extrapolated from the medical documentation and two patients were missing to follow-up.

## Conclusions

Due to the rarity and recentness of NORSE, data about diagnosis, treatment and outcome for this disease are scarce. Until reliable prognostic scores are available, decisions to limit treatment for these young and previously healthy patients should be taken very carefully. Our data suggest that a favorable long-term outcome is still possible, despite complicated and long hospital stays.

## Data Availability

Will be provided on reasonable request.

## Abbreviations

NORSE: New onset refractory status epilepticus
ICU: Intensive care unit
SE: Status epilepticus
EOL: End of life
NCCU: Neurocritical Care Unit
mRS: Modified Rankin scale
AED: Antiepileptic drugs
MRI: Magnetic resonance imaging
LST: Life-sustaining therapies
SDM: Surrogate decision makers
